# A primary rodent triculture model to investigate the role of glia-neuron crosstalk in regulation of neuronal activity

**DOI:** 10.3389/fnagi.2022.1056067

**Published:** 2022-12-01

**Authors:** Leela Phadke, Dawn H. W. Lau, Nozie D. Aghaizu, Shania Ibarra, Carmen M. Navarron, Lucy Granat, Lorenza Magno, Paul Whiting, Sarah Jolly

**Affiliations:** ^1^Alzheimer’s Research UK Drug Discovery Institute, University College London, London, United Kingdom; ^2^UK Dementia Research Institute, University College London, London, United Kingdom

**Keywords:** drug discovery, microglia, astrocytes, neuroinflammation, hyperexcitability, triculture, multi-electrode array

## Abstract

Neuroinflammation and hyperexcitability have been implicated in the pathogenesis of neurodegenerative disease, and new models are required to investigate the cellular crosstalk involved in these processes. We developed an approach to generate a quantitative and reproducible triculture system that is suitable for pharmacological studies. While primary rat cells were previously grown in a coculture medium formulated to support only neurons and astrocytes, we now optimised a protocol to generate tricultures containing neurons, astrocytes and microglia by culturing in a medium designed to support all three cell types and adding exogenous microglia to cocultures. Immunocytochemistry was used to confirm the intended cell types were present. The percentage of ramified microglia in the tricultures decreases as the number of microglia present increases. Multi-electrode array recordings indicate that microglia in the triculture model suppress neuronal activity in a dose-dependent manner. Neurons in both cocultures and tricultures are responsive to the potassium channel blocker 4-aminopyridine, suggesting that neurons remained viable and functional in the triculture model. Furthermore, suppressed neuronal activity in tricultures correlates with decreased densities of dendritic spines and of the postsynaptic protein Homer1 along dendrites, indicative of a direct or indirect effect of microglia on synapse function. We thus present a functional triculture model, which, due to its more complete cellular composition, is a more relevant model than standard cocultures. The model can be used to probe glia-neuron interactions and subsequently aid the development of assays for drug discovery, using neuronal excitability as a functional endpoint.

## Introduction

Alzheimer’s disease (AD) is the most common cause of dementia, accounting for an estimated 60–80% of cases worldwide (Alzheimer’s Association, 2020). The two main pathological hallmarks of AD in the brain are amyloid-β (Aβ) plaques and hyperphosphorylated tau aggregates known as neurofibrillary tangles (NFTs). Both Aβ and phosphorylated tau are known to be early biomarkers of AD, and it has been proposed that Aβ accumulation initiates a cascade of events that ultimately results in neuronal damage (Palmqvist et al., 2019). However, the linearity of this cascade remains controversial and accumulating evidence suggests that many processes are involved in the pathogenesis of AD (De Strooper and Karran, 2016). Neuronal hyperexcitability has been implicated in the pathogenesis of neurodegenerative disease and correlates with cognitive decline in patients with AD (Vossel et al., 2013; Harris et al., 2020). In addition, studies have found that elevated levels of inflammatory markers are present in patients with AD (Brosseron et al., 2018), and a number of AD risk genes are associated with innate immune functions (Efthymiou and Goate, 2017; Bellenguez et al., 2022). Therefore, neuroinflammation is thought to play a significant role in the pathogenesis of the disease and the immune cells involved in this process are astrocytes and microglia. Both cell types undergo morphological and molecular remodelling in ageing and disease, and display a wide repertoire of functional states (Escartin et al., 2021; Paolicelli et al., 2022). Astrocytes are the most abundant cell type in the central nervous system (CNS). Their functions include the maintenance of neurotransmitter homeostasis, synapse formation and metabolic and neurotrophic support for neurons (Rouach et al., 2008; Eroglu and Barres, 2010). Astrocytes are known to respond to pathological insults through reactive gliosis, which involves morphological, molecular, and functional remodelling, and they can also become atrophic, showing reduced volume and a reduced number of processes (Arranz and De Strooper, 2019; Escartin et al., 2021). Microglia are the resident macrophages of the CNS that play important roles in synaptic pruning, neuronal apoptosis, and maintenance of synaptic plasticity (Schafer et al., 2012; Weinhard et al., 2018; York et al., 2018). Microglia have also been shown to respond to neuronal activation by suppressing neuronal activity (Badimon et al., 2020). Homeostatic, ramified microglia constantly survey the CNS with their processes, sensing damage signals and mediating a response toward the site of injury (Nimmerjahn et al., 2005). Microglia *in vivo* are highly dynamic and heterogeneous, allowing them to achieve a range of responses depending on the environmental context. It remains unclear whether microglial function in neurodegenerative disease is beneficial but insufficient, or whether they are effective in early disease before losing their efficacy or becoming detrimental (Masuda et al., 2020).

Analysis of tissue from the mouse CNS during homeostasis reveals specific time- and region-dependent subtypes of microglia, and additional context-dependent subtypes are found in mouse models of neurodegeneration and demyelination. These subtypes correspond to similar clusters identified in healthy human brains and the brains of multiple sclerosis patients (Masuda et al., 2019). This heterogeneity is difficult to capture in a classically used monoculture system. Developing more complex cultures with other cell types combined in the same environment may provide a better model of the diverse phenotypic states observed *in vivo*. Neurons, astrocytes and microglia act in a synchronised manner, and communication between all these cell types can be disrupted by, and contribute to, neurodegenerative diseases. For example, in the presence of Aβ, microglia become responsive to interleukin-3 (IL-3) produced by astrocytes and undergo transcriptional and phenotypic changes leading them to a more reactive state that can restrict AD pathology (McAlpine et al., 2021). Aβ was also shown to activate the nuclear factor kappa-light-chain-enhancer of activated B cells (NF-κB) pathway in astrocytes and increase the release of complement component 3 (C3), resulting in neuronal dysfunction and microglial activation via C3a receptor signalling (Lian et al., 2015). Conversely, activated microglia induce a specific state of reactive astrocytes by secreting interleukin-1 alpha (IL-1α), tumour necrosis factor (TNF) and complement component 1q (C1q) and this population of astrocytes has been shown to be neurotoxic (Liddelow et al., 2017; Guttenplan et al., 2021). Furthermore, selective blocking of Aβ-induced microglial activation via glucagon-like peptide-1 receptor (GLP-1R) activation inhibits formation of reactive astrocytes and preserves neuronal viability in AD models (Park et al., 2021). Dysregulated neuronal-glial crosstalk has also been observed in human AD. Under physiological conditions, neuronal-microglial crosstalk via CD200–CD200 receptor (CD200R) and fractalkine (CX3CL1)–CX3C motif chemokine receptor 1 (CX3CR1) signalling is thought to regulate the homeostatic function of microglia (Simon et al., 2019). In the brains of AD patients, however, expression of CD200, CD200R, and CX3CR1 is reduced, suggesting a loss of physiological constraints on microglial function (Walker et al., 2009).

There is a need for more complex *in vitro* systems able to model the crosstalk between different cell types in the CNS. Cocultures of neurons and astrocytes are an established method of studying neuroinflammation *in vitro* but these models are not able to capture the interactions with microglia or how both astrocytes and microglia may affect neuronal activity. We thus generated an experimental rat triculture platform comprised of all three major cell types associated with neuroinflammation (neurons, astrocytes, and microglia) that is quantitative and reproducible to allow for pharmacological studies and screening. Primary rat cells were maintained in a serum-free culture media that we optimised to support all three cell types. To validate our platform, we utilised immunostaining followed by high content imaging and multi-electrode array (MEA) as endpoints. MEA technology is a powerful tool for quantifying the activity of neuronal networks in a fast and non-invasive manner. We showed that this triculture model can be maintained for at least 17 days *in vitro* (DIV17) and that microglia in the model do not affect the number of neurons but suppress neuronal activity in a dose-dependent manner. This suppression of neuronal activity correlates with decreased densities of dendritic spines and of the postsynaptic protein Homer1 along dendrites. Here we developed an assay for investigating glia-mediated regulation of cortical neuron activity, which is amenable to pharmacological modulation. This platform will facilitate drug discovery by enabling target identification studies as well as drug screening.

## Materials and methods

### Animals

All procedures involving animals were conducted according to the UCL Ethics Committee guidelines and the UK Animals Scientific Procedures Act UK (1986) and its Amendment Regulations (2012). Wildtype Sprague-Dawley rats (Charles River, Wilmington, USA) were used in this study.

### Primary cortical neuron and astrocyte coculture

Cocultures were prepared from the neocortex of embryonic day 18 (E18) embryos of Sprague-Dawley rats. Meninges were removed and the cerebral cortices were dissected. The tissue was then incubated with 0.25% trypsin-EDTA (25200056, Gibco, Waltham, USA) and 1 mg/ml DNase I (DN25, Sigma-Aldrich, St. Louis, USA) for 10 min at 37°C. The cells were mechanically dissociated in DMEM (31966021, Gibco, Waltham, USA) complemented with DNase I and washed twice in DMEM. 25,000 neurons/well were plated on a 96-well imaging plate (6055300, Perkin-Elmer, Waltham, USA) coated overnight with Poly-D-Lysine (PDL) (P0899, Sigma-Aldrich, St. Louis, USA), and 50,000 neurons/well were plated on a 96-well MEA plate (see MEA Methods for detailed protocol). The cultures were maintained for 24 h in a plating media composed of DMEM supplemented with Glutamax 1x (35050061, Gibco, Waltham, USA), 5% horse serum (26050088, Gibco, Waltham, USA), 1 mM L-glutamine (25030024, Gibco, Waltham, USA), and penicillin/streptomycin (15140148, Gibco, Waltham, USA). On DIV1, medium was removed and replaced with maintenance media composed of Neurobasal (21103049, Gibco, Waltham, USA), B27 1x (17504044, Gibco, Waltham, USA), N2 1x (17502048, Gibco, Waltham, USA), 1 mM L-glutamine, Glutamax 1x, and penicillin/streptomycin. Twice a week, half of the medium was removed and replaced with fresh medium. The cultures were maintained in a humidified incubator at 37°C under an atmosphere containing 5% CO_2_. Cocultures were used for experimentation between DIV13 and DIV17.

### Primary cortical neuronal culture

Neuronal cultures were initially prepared as above, using the same method as the cocultures. Cultures were maintained for 4 h in plating media before medium was removed and replaced with fresh maintenance media. Twice a week, half of the medium was removed and replaced with fresh medium. On DIV6, medium was supplemented with CultureOne Supplement (A3320201, Gibco, Waltham, USA). The cultures were maintained in a humidified incubator at 37°C under an atmosphere containing 5% CO_2_.

### Primary microglia culture

Microglia were prepared from postnatal day 14 (P14) Sprague-Dawley rats. The meninges, cerebellum and olfactory bulbs were removed before the hemispheres were minced into small pieces using a sharp blade. Minced tissue was mechanically dissociated in a glass homogeniser in phosphate buffered saline (PBS) (14190094, Gibco, Waltham, USA), centrifuged at 1,380 g for 10 min and the pellet resuspended in 70% isotonic Percoll (P1644, Sigma-Aldrich, St. Louis, USA). Layers of 30% isotonic Percoll and PBS were slowly added before centrifuging at 1,090 g for 50 min with slow acceleration/deceleration. The myelin layer was discarded from the top of the Percoll gradient, and the microglia collected from the interface between the 70% and 30% Percoll layers. Microglia were diluted in PBS and centrifuged at 1,570 g for 10 min and the pellet resuspended in DMEM/F12 (21041025, Gibco, Waltham, USA) containing penicillin/streptomycin, 2 mM L-glutamine, 5 μg/ml *N*-acetyl cysteine (A8199, Sigma-Aldrich, St. Louis, USA), 5 μg/ml insulin (I6634, Sigma-Aldrich, St. Louis, USA), 100 μg/ml apo-transferrin (T1147, Sigma-Aldrich, St. Louis, USA), and 100 ng/ml sodium selenite (S5261, Sigma-Aldrich, St. Louis, USA) (basal media) supplemented with 2 ng/ml transforming growth factor-beta 2 (TGF-β2) (100-35B, Peprotech, Cranbury, USA), 100 ng/ml interleukin-34 (IL-34) (577606, BioLegend, San Diego, USA), 1.5 μg/ml cholesterol (700000P, Sigma-Aldrich, St. Louis, USA), and 1 μg/ml heparan sulfate (AMS.GAG-HS01, Amsbio, Abingdon, UK) (TICH media). Cultures were maintained in a humidified incubator at 37°C under an atmosphere containing 5% CO_2_.

### Primary neuron–astrocyte–microglia triculture

**FIGURE 1:**
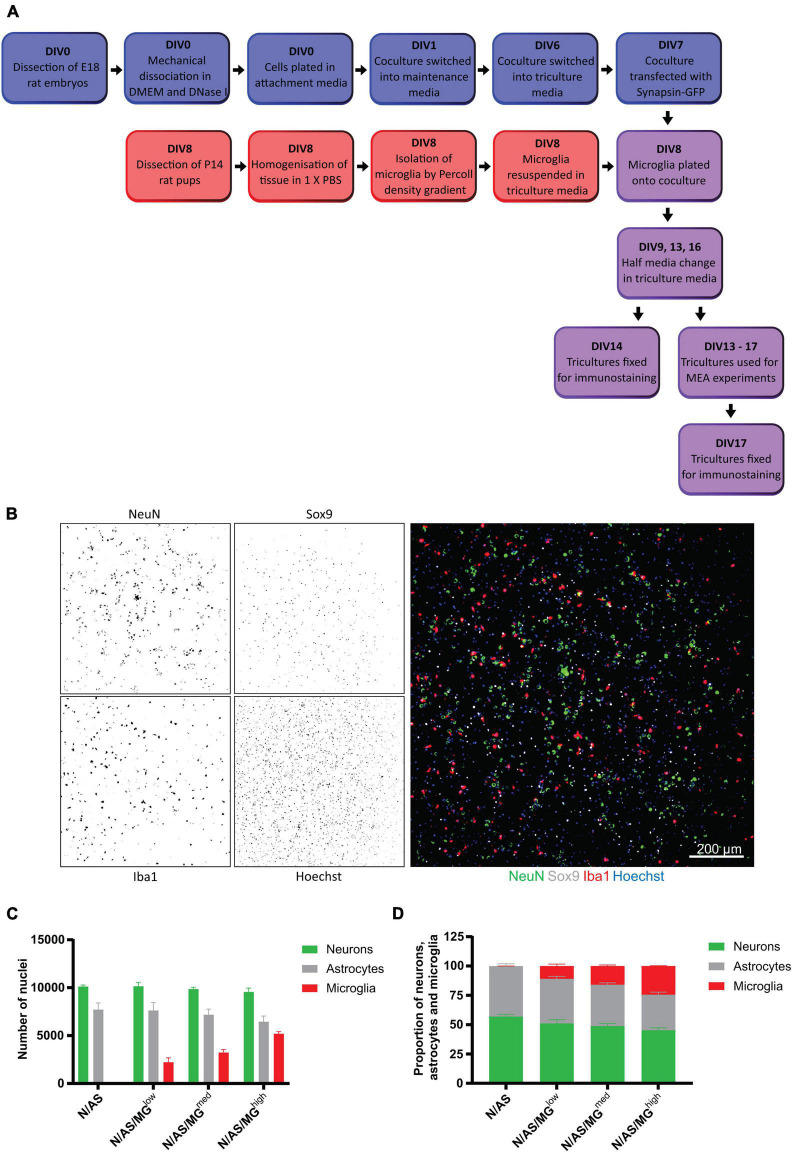
Development of a triculture system with rat neurons, astrocytes, and microglia. **(A)** Schematic workflow of neurons, astrocytes, and microglia plating, maintenance, and maturation. Blue = coculture of neurons and astrocytes. Red = microglia. Purple = triculture of neurons, astrocytes, and microglia. Mature cultures (DIV13+) were used for various experimental treatments and conditions. At the end of the experiments, fixed cells were processed for immunostaining. **(B–D)** Quantification of cell numbers by immunocytochemistry. **(B)** Representative immunostaining image used for quantification showing NeuN (neuronal marker), Sox9 (astrocyte marker), and Iba1 (microglia marker) expression at DIV14 in triculture N/AS/MG^high^. Merge shows staining of NeuN (green), Sox9 (white), Iba1 (red), and Hoechst (blue). Scale bar = 200 μm. **(C)** Cell count analysis of the total number of cells per well performed on cocultures and tricultures at DIV14 using Harmony software. There was no significant difference in astrocyte populations in the tricultures compared to the coculture (N/AS/MG^low^ *p* > 0.9999, N/AS/MG^med^ *p* = 0.9993, N/AS/MG^high^ *p* = 0.7218, 2-way ANOVA, *post hoc* Tukey’s multiple comparisons test). Data shown as mean ± SEM and is representative of three separate experiments. **(D)** Cell count analysis shown as proportions of the total number of neurons, astrocytes, and microglia. Data shown as mean ± SEM and is representative of three independent experiments.

Cocultures were prepared as above and cultured in maintenance media until DIV6, from which point cells were cultured in maintenance media supplemented with 2 ng/ml TGF-β2, 100 ng/ml IL-34, and 1.5 μg/ml cholesterol (triculture media). On DIV8, microglia were isolated and centrifuged at 1,570 g for 7 min to remove the TICH media. The pellet was resuspended in triculture media and 20 μl of microglia was added to the coculture in neuron/astrocyte:microglia ratios of 1:0.1, 1:0.3 and 1:1.5. Twice a week, half of the medium was removed and replaced with fresh medium. Cultures were maintained in a humidified incubator at 37°C under an atmosphere containing 5% CO_2_. Tricultures were used for experimentation between DIV13 and DIV17 (Figure 1A).

### Transfection

Neuronal transfection with the DNA construct pHR hsyn:EGFP [Keaveney et al., 2018; kind gift from Xue Han^1^ (Addgene plasmid #114215; RRID:Addgene_114215)] was performed at DIV7 using the Neuromag magnetofection method (OzBiosciences, Marseille, France) (Figure 1A). For each well of a 96-well plate, 1.5 μg DNA was diluted in 100 μl of Opti-MEM (11058021, Gibco, Waltham, USA) and added dropwise to 1 μl Neuromag transfection reagent, with all reagents at room temperature. Following 20 min incubation at room temperature, the transfection mix was added dropwise to neuronal cultures and the 96-well plate was placed on a magnetic plate (OzBiosciences, Marseille, France) pre-equilibrated to 37°C inside an incubator. After 15 min of magnetofection, the culture plate was removed from the magnetic plate and normal cell culture resumed.

### Immunocytochemistry

Cultures fixed in 4% paraformaldehyde (PFA) (F8775, Sigma-Aldrich, St. Louis, USA) were labelled after membrane permeabilisation and saturation with 10% fetal bovine serum (FBS) (F9665, Sigma-Aldrich, St. Louis, USA) and 0.02% Triton X-100 (X100, Sigma-Aldrich, St. Louis, USA) in PBS for 1 h. Cells plated for imaging were incubated with mouse NeuN (1:500, ab104224, Abcam, Cambridge, UK), rabbit SRY-box transcription factor 9 (Sox9) (1:500, ab185230, Abcam, Cambridge, UK), guinea pig ionised calcium-binding adapter molecule 1 (Iba1) (1:500, 234 004, Synaptic Systems, Goettingen, Germany) and rabbit oligodendrocyte transcription factor 2 (Olig2) (1:500, ab109186, Abcam, Cambridge, UK). Cells plated in MEA plates were incubated with mouse NeuN (1:250), rabbit Sox9 (1:250) and guinea pig Iba1 (1:500). Antibodies were diluted in in 10% FBS and 0.02% Triton X-100 in PBS and incubated overnight at 4°C. After rinsing three times in PBS, cells were incubated with anti-mouse Alexa Fluor 488 (A21202, Invitrogen, Waltham, USA), anti-guinea pig Alexa Fluor 568 (ab175714, Abcam, Cambridge, UK) and anti-rabbit Alexa Fluor 647 (A21443, Invitrogen, Waltham, USA) diluted 1:1,000 in PBS with 10% FBS and 0.02% Triton X-100 for 2 h at room temperature. Nuclei were stained with Hoechst 33258 (1:1,000, 94403, Sigma-Aldrich, St. Louis, USA) and cells washed three times in PBS.

For synaptic staining, transfected cultures fixed in 4% PFA and 4% sucrose (S0389, Sigma-Aldrich, St. Louis, USA) were labelled after membrane permeabilisation and saturation with 5% normal goat serum (NGS) (C07SA, BioRad, Hercules, USA), 1% bovine serum albumin (BSA) (B6917, Sigma-Aldrich, St. Louis, USA), and 0.1% Triton X-100 in PBS for 1 h. Cells were incubated with rabbit Homer1 (1:500, 160 003, Synaptic Systems, Goettingen, Germany) and guinea pig vesicular glutamate transporter 1 (VGlut1) (1:300, AB5905, Sigma-Aldrich, St. Louis, USA). Antibodies were diluted in 5% NGS, 1% BSA, and 0.1% Triton X-100 in PBS and incubated overnight at 4°C. After rinsing three times in PBS, cells were incubated with anti-rabbit Alexa Fluor 568 (A10042, Invitrogen, Waltham, USA) and anti-guinea pig Alexa Fluor 647 (ab150187, Abcam, Cambridge, UK) diluted 1:500 in PBS with 5% NGS, 1% BSA, and 0.1% Triton X-100 for 2 h at room temperature. Nuclei were stained with Hoechst 33258 (1:1,000) and cells washed three times in PBS.

### Image acquisition

For cultures used for cell quantification, images were acquired using the Opera Phenix High-Content Screening System (Perkin-Elmer, Waltham, USA). Nine fields of view were taken at 10× magnification to scan an entire 96-well culture well. To analyse the morphology of microglia in the tricultures, images were again acquired using the Opera Phenix High-Content Screening System. Nine fields of view were taken at 20× magnification to obtain representative images spread across each 96-well culture well.

For cultures on MEA plates, images were acquired using the Celldiscoverer 7 Automated Microscope (Zeiss, Oberkochen, Germany). Thirty-six fields of view were taken at 5× magnification to scan an entire 96-well culture well.

For transfected cultures used for synaptic staining, images were acquired using the LSM880 Confocal (Zeiss, Oberkochen, Germany). Whole neurons were acquired using a 40× objective (NA = 1.3), at a resolution of 1,024 × 1,024 pixels, a step size of 1 μm, and with photomultiplier tubes to detect fluorescence emission. Secondary dendrites were acquired at a higher magnification using the 63× objective (NA = 1.4) with an additional 3.5× zoom at a resolution of 1,024 × 1,024 pixels and at a step size of 0.5 μm. A total of six neurons and accompanying secondary dendrites spread across three biological repeats were imaged per condition.

### Image analysis

The Harmony High-Content Imaging and Analysis Software (Perkin-Elmer, Waltham, USA) was used to quantify the neuron, astrocyte and microglia cell numbers in culture. Microglia were classified into either amoeboid or ramified phenotypes using the PhenoLOGIC machine-learning algorithm within the Harmony software, with modifications to the training of the algorithm to enable recognition of microglia within the tricultures.

Dendritic spine and synapse analysis on hSyn:EGFP expressing cortical neurons was performed using Imaris software (Oxford Instruments, Abingdon, UK). Briefly, the “Filament” tool was used to semi-automatically specify the secondary dendrite within an image file, followed by the detection of dendritic spines by manual identification. The green fluorescent protein (GFP) signal was used to create an exclusion mask using the “Surface” tool to isolate the Homer1 signal within a dendrite of interest. Homer1 puncta were subsequently identified using the “Spot” detection tool set to a detection diameter of 0.45 μm. Background Homer signal was excluded by thresholding using the “Quality” filter. Pre-synaptic vGlut1 puncta in the entire image were similarly identified using the “Spot” detection tool at 0.45 μm diameter. Synapses were assumed using the “Co-localise spots” function within the “Spot” detection tool when there was a maximum distance of 1 μm between Homer1 and vGlut1 puncta.

### Multi-electrode array

Each well of a 96-well MEA plate (M768-tMEA-96B, Axion Biosystems, Atlanta, USA) was spot-coated with 8 μl of polyethylenimine (PEI) (P3143, Sigma-Aldrich, St. Louis, USA) solution (0.2% PEI in 0.1 M sodium borate pH 8.4) and plates were incubated at room temperature overnight. Wells were washed three times with water before being left to dry under the biological safety cabinet for approximately 1 h. Wells were then spot-coated with 8 μl of laminin (20 μg/ml, L2020, Sigma-Aldrich, St. Louis, USA). Sterile deionised water was added to the area surrounding the wells to prevent substrate evaporation and the plates were incubated for 1 h at 37°C without letting the laminin droplet dry. Laminin was removed directly before seeding the well with neurons. 50,000 neurons/well were spot-coated in 8 μl of plating media. Ten replicates were plated per condition in the inner wells of the plate. Plates were incubated for 1 h at 37°C before 150 μl of plating media was added to each well. On the next day cells were transferred in maintenance media and maintained as described above. Electrical activity of neurons was recorded daily between DIV13 and DIV17. When recordings took place on the same day as the half-media changes, neurons were recorded prior to changing the media as changes will temporarily alter neuronal activity. On the recording day, the plate was loaded into the Axion Maestro MEA reader (Axion Biosystems, Atlanta, USA), let to equilibrate for 10 min and recording was performed via AxIS for 15 min (1,000 X Gain, 200 Hz–3 kHz). Data was first analysed with AxIS software (Spike Detector Threshold 6 × Std Dev) and then subsequently using GraphPad Prism 9 software (GraphPad Software, San Diego, USA).

### Drug treatment

On DIV17, half an hour after basal neuronal activity was recorded, the MEA plate was treated by removing half of the medium and replacing with medium containing 100 μM 4-Aminopyridine (4-AP) (275875, Sigma-Aldrich, St. Louis, USA) at 2X. Recordings were performed 1 h after treatment.

For analysis, wells were excluded using the ROUT method to detect outliers (*Q* = 10%) in GraphPad Prism 9.

### Statistical analysis

All mean values are presented and stated as ±standard error of mean (SEM). Statistical analyses were performed using GraphPad Prism 9. Data normality was tested by the Shapiro-Wilk test.

Comparisons between two groups were analysed by unpaired *t*-test. One-way ANOVA was used for comparisons between >2 groups in datasets with one variable. Two-way ANOVA was used for comparisons between >2 groups in datasets with two variables. *Post hoc* Tukey’s multiple comparisons test was used after performing a one- or two-way ANOVA.

## Results

### Generation of a triculture model with primary rat neurons, astrocytes, and microglia

Our aim was to establish a triculture model allowing for the investigation of glial contribution to neuronal activity. Primary cortical cells from E18 rat embryos were cultured in neuron-astrocyte coculture media for 6 days (Figure 1A). Early experiments showed that when microglia were added to the culture at DIV8 and cultured in coculture media, less than 1% microglia were present by DIV14 (data not shown). TGF-β2, IL-34, and cholesterol have been identified as the factors essential for microglial survival in serum-free, defined medium conditions. Microglia in isolation can be cultured in a serum-free “TICH” media supplemented with TGF-β2, IL-34, and cholesterol as well as heparan sulfate, which improves cell adhesion and process extension (Bohlen et al., 2017). Microglia were initially plated on cocultures in TICH media but experiments showed that this addition of microglia or addition of TICH media alone caused a significant reduction in the number of NeuN-positive neuronal nuclei present (data not shown). To support microglial survival and avoid the neurotoxicity due to the addition of microglia in TICH media, we developed a triculture media supporting all three cell types, which was composed of the coculture media supplemented with TGF-β2, IL-34, and cholesterol.

To allow the neurons and astrocytes to adjust to the additional media components prior to addition of the microglia on DIV8, cocultures were switched into triculture media 48 h before on DIV6. Exogenous microglia isolated from P14 rats were resuspended in triculture media and plated onto the coculture on DIV8 at three different ratios: 1 neuron/astrocyte:0.1 microglia, 1 neuron/astrocyte:0.3 microglia, and 1 neuron/astrocyte:1.5 microglia, referred to as N/AS/MG*^low^*, N/AS/MG*^med^*, and N/AS/MG*^high^*, respectively (Figure 1A). These ratios were chosen to cover the range of cell densities that have been reported in the human brain, as the exact numbers remain unknown and appear to vary between different brain regions (Herculano-Houzel, 2014; von Bartheld et al., 2016). Iba1 is a protein expressed specifically by microglia in the brain (Ito et al., 1998). Immunostaining for Iba1 showed that at DIV14 there was a population of microglia present in the triculture which increased dose-dependently and was absent in the coculture (Figures 1B,C). Immunostaining for the neuronal marker NeuN and the astrocyte-specific nuclear marker Sox9 indicated a healthy population of neurons and astrocytes in both the cocultures and tricultures (Figures 1B,C). The number of neurons was not affected by the presence of the microglia (Figure 1C). While the analysis also did not reveal a significant difference in astrocyte populations in the tricultures compared to the coculture (N/AS/MG*^low^ p* > 0.9999, N/AS/MG*^med^ p* = 0.9993, N/AS/MG*^high^ p* = 0.7218), the results suggest that the N/AS/MG*^high^* triculture may contain slightly lower numbers of astrocytes than the coculture (three independent biological repeats were quantified). As a proportion of the total number of neurons, astrocytes and microglia in culture, cocultures contained 56.89 ± 1.952% neurons, 43.10 ± 1.952% astrocytes and no microglia. The N/AS/MG*^low^* triculture contained 51.13 ± 3.133% neurons, 37.98 ± 2.031% astrocytes, and 10.89 ± 1.5665% microglia. The N/AS/MG*^med^* triculture contained 48.82 ± 2.279% neurons, 35.32 ± 1.527% astrocytes, and 15.89 ± 0.8791% microglia. The N/AS/MG*^high^* triculture contained 45.22 ± 2.169% neurons, 30.33 ± 2.192 astrocytes, and 24.45 ± 0.2614% microglia (Figure 1D). Note that the NeuN staining was sometimes weak and/or did not mark all the neurons. Additional staining for Olig2 (Valério-Gomes et al., 2018) revealed that both cocultures and tricultures contained 3–8% of cells from the oligodendrocyte lineage (data not shown).

**FIGURE 2:**
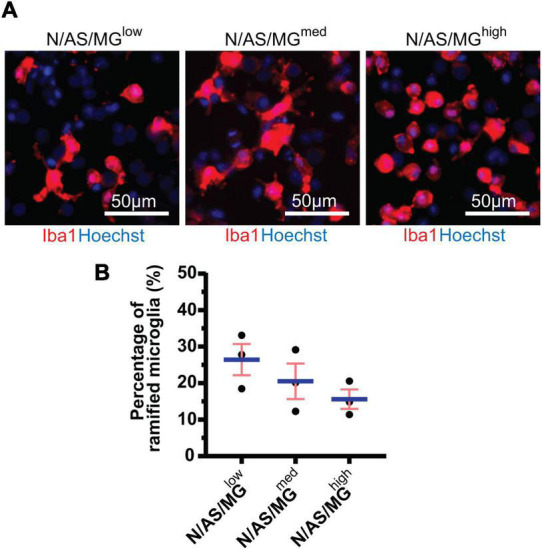
Microglia in tricultures with higher numbers of microglia display a less ramified morphology than tricultures with less microglia. **(A)** Representative immunostaining images used for morphology analysis showing Iba1 expression at DIV14 in tricultures N/AS/MG^low^, N/AS/MG^med^, and N/AS/MG^high^. Merge shows staining of Iba1 (red) and Hoechst (blue). Scale bar = 50 μm. **(B)** Morphology analysis performed on Iba1-positive cells in tricultures at DIV14 using Harmony software. The difference between the three triculture conditions is not statistically significant (*p* = 0.2440, one-way ANOVA). However, the difference between the percentage of ramified microglia in the N/AS/MG^low^ triculture and the N/AS/MG^high^ triculture (*p* = 0.2201, one-way ANOVA, *post hoc* Tukey’s multiple comparisons test) is larger than the difference between the N/AS/MG^low^ triculture and the N/AS/MG^med^ triculture (*p* = 0.5839, one-way ANOVA, *post hoc* Tukey’s multiple comparisons test). Data shown as mean ± SEM and is representative of three independent experiments.

Microglia *in vitro* display a high degree of heterogeneity in terms of morphology. Homeostatic microglia display ramifications and processes, whereas activated microglia are more amoeboid. As microglial morphology gives an indication as to the phenotypic state of the cell, we performed further analysis on the tricultures to classify the morphology of microglia as either ramified or amoeboid. While the difference between the three triculture conditions is not statistically significant (*p* = 0.2440), the trend indicates that the percentage of ramified microglia present in the triculture decreases dose-dependently with increasing numbers of microglia. The difference between the percentage of ramified microglia in the N/AS/MG*^low^* triculture and the N/AS/MG*^high^* triculture (*p* = 0.2201) is larger than the difference between the N/AS/MG*^low^* triculture and the N/AS/MG*^med^* triculture (*p* = 0.5839; Figures 2A,B). The average percentage of ramified microglia from three biological repeats was 26.45 ± 4.272% for the N/AS/MG*^low^* triculture, 20.53 ± 4.875% for the N/AS/MG*^med^* triculture, and 15.61 ± 2.668% for the N/AS/MG*^high^* triculture (Figure 2B).

### Use of multi-electrode array to measure neuronal activity shows that microglia suppress spontaneous neuronal activity

**FIGURE 3:**
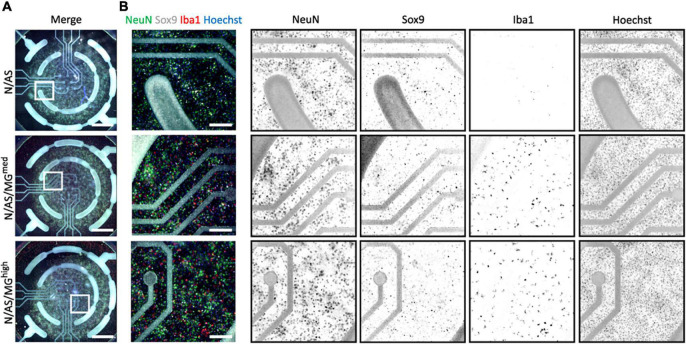
Cocultures and tricultures of rat cells are cultured on multi-electrode array (MEA) plates to record neuronal activity. **(A)** Representative whole-well immunostaining images showing NeuN, Sox9, and Iba1 expression in cocultures and tricultures used for spontaneous activity recordings in MEA plates at DIV17. Merge shows staining of NeuN (green), Sox9 (white), Iba1 (red), and Hoechst (blue). Scale bar = 1,000 μm. White frame = field of view of image. **(B)** Representative snapshot immunostaining images showing NeuN, Sox9, and Iba1 expression in cocultures and tricultures used for spontaneous activity recording in MEA plates. Merge shows staining of NeuN (green), Sox9 (white), Iba1 (red), and Hoechst (blue). Scale bar = 200 μm.

**FIGURE 4:**
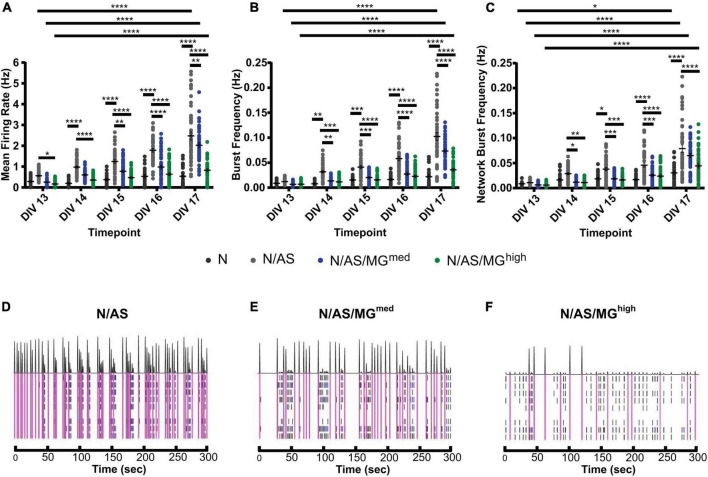
Microglia suppress spontaneous neuronal activity in a dose-dependent manner. **(A–C)** Spontaneous neuronal activity in neuronal cultures, cocultures, and tricultures was recorded daily from DIV13 to DIV17. **(A)** Mean firing rate was higher in cocultures compared to neuronal cultures at all time points recorded after DIV13 (DIV14 to DIV17 *p* < 0.0001, 2-way ANOVA, *post hoc* Tukey’s multiple comparisons test). Mean firing rate was higher in cocultures compared to triculture N/AS/MG^high^ at all time points recorded (DIV13 *p* = 0.0456, DIV14 to DIV17 *p* < 0.0001, 2-way ANOVA, *post hoc* Tukey’s multiple comparisons test). Mean firing rate was higher in cocultures compared to triculture N/AS/MG^med^ at all time points recorded after DIV14 (DIV15 *p* = 0.0024, DIV16 *p* < 0.0001, DIV17 *p* = 0.0048, 2-way ANOVA, *post hoc* Tukey’s multiple comparisons test). Mean firing rate was higher at DIV17 compared to DIV13 in both the cocultures and tricultures (*p* < 0.0001, 2-way ANOVA, *post hoc* Tukey’s multiple comparisons test). **(B)** Burst frequency was higher in cocultures compared to neuronal cultures at all time points recorded after DIV13 (DIV14 *p* = 0.002, DIV15 *p* = 0.0002, DIV16 to DIV17 *p* < 0.0001, 2-way ANOVA, *post hoc* Tukey’s multiple comparisons test). Burst frequency was higher in cocultures compared to triculture N/AS/MG^high^ at all time points recorded after DIV13 (DIV14 *p* = 0.003, DIV15 to DIV17 *p* < 0.0001, 2-way ANOVA, *post hoc* Tukey’s multiple comparisons test). Burst frequency was higher in cocultures compared to triculture N/AS/MG^med^ at all time points recorded after DIV13 (DIV14 *p* = 0.0023, DIV15 *p* = 0.0001, DIV16 to DIV17 *p* < 0.0001, 2-way ANOVA, *post hoc* Tukey’s multiple comparisons test). Burst frequency was higher at DIV17 compared to DIV13 in both the cocultures and tricultures (*p* < 0.0001, 2-way ANOVA, *post hoc* Tukey’s multiple comparisons test). **(C)** Network burst frequency was higher in cocultures compared to neuronal cultures at all time points recorded after DIV14 (DIV15 *p* = 0.0318, DIV16 to DIV17 *p* < 0.0001, 2-way ANOVA, *post hoc* Tukey’s multiple comparisons test). Network burst frequency was higher in cocultures compared to triculture N/AS/MG^high^ at all time points recorded after DIV13 (DIV14 *p* = 0.0058, DIV15 *p* = 0.0001, DIV16 *p* < 0.0001, DIV17 *p* < 0.0001, 2-way ANOVA, *post hoc* Tukey’s multiple comparisons test). Network burst frequency was higher in cocultures compared to triculture N/AS/MG^med^ at all time points recorded between DIV14 and DIV16 (DIV14 *p* = 0.0112, DIV15 *p* = 0.0007, DIV16 *p* = 0.0004, 2-way ANOVA, *post hoc* Tukey’s multiple comparisons test). Network burst frequency was higher at DIV17 compared to DIV13 in both the cocultures and tricultures (*p* < 0.0001, 2-way ANOVA, *post hoc* Tukey’s multiple comparisons test). Network burst frequency was also higher at DIV17 compared to DIV13 in neuronal cultures (*p* = 0.0483, 2-way ANOVA, *post hoc* Tukey’s multiple comparisons test). **(D–F)** Representative activity traces at DIV17 from coculture N/AS and tricultures N/AS/MG^med^ and N/AS/MG^high^. Black spikes at the top represent network activity while activity over time recorded on each of the eight electrodes is shown under. Blue = bursts. Pink = network bursts. Data shown as mean ± SEM and is representative of five independent experiments.

As both microglia and astrocytes can regulate neuronal activity in basal conditions as well as in disease, we set up an assay allowing for the assessment of glia-mediated regulation of cortical neuron activity. MEA is a high throughput assay allowing recording of spontaneous neuronal activity over time in a non-invasive way. Following optimisation of the protocol, neuronal cultures, cocultures and tricultures were cultured on 96-well MEA plates to record neuronal activity (see “Materials and methods” for detailed protocol). Neuronal cultures and cocultures are plated by spot-coating onto the centre of the electrodes. Upon their addition, microglia attach to the whole well, not only the electrodes (Figures 3A,B). Therefore, the final ratios of the tricultures in the MEA plates were lower than the same ratios plated onto imaging plates. The N/AS/MG*^med^* and N/AS/MG*^high^* tricultures were chosen for experimentation on the MEA to increase the likelihood that the final ratio was sufficient to characterise the effect of the microglia on neuronal activity. Immunostaining for NeuN, Sox9, and Iba1 confirmed that the intended cell types were present at DIV17 in the cocultures and tricultures cultured on MEA plates (Figures 3A,B). Spontaneous neuronal activity in neuronal cultures, cocultures, and tricultures from five independent biological repeats was recorded daily from DIV13 to DIV17. The software identified action potentials or “spikes”, clusters of spikes referred to as bursts, and coordinated clusters of spikes across multiple electrodes referred to as network bursts. The network burst frequency significantly increased from DIV13 to DIV17 in neuronal cultures (Figure 4C, *p* = 0.0483), with the mean firing rate and burst frequency displaying a similar albeit not significant trend. The mean firing rate, burst frequency and network burst frequency significantly increased from DIV13 to DIV17 in the cocultures and both tricultures (Figures 4A–C, Mean firing rate *p* < 0.0001, Burst frequency *p* < 0.0001, Network burst frequency *p* < 0.0001). The mean firing rate was significantly lower in neuronal cultures compared to cocultures at all time points recorded after DIV13 (Figure 4A, DIV14 to DIV17 *p* < 0.0001). Burst frequency was significantly lower in neuronal cultures compared to cocultures at all time points recorded after DIV13 (Figure 4B, DIV14 *p* = 0.002, DIV15 *p* = 0.0002, DIV16 to DIV17 *p* < 0.0001). Network burst frequency was significantly lower in neuronal cultures compared to cocultures at all time points recorded after DIV14 (Figure 4C, DIV15 *p* = 0.0318, DIV16 to DIV17 *p* < 0.0001). Microglia suppressed spontaneous neuronal activity in a dose-dependent manner. The mean firing rate was significantly lower in triculture N/AS/MG*^high^* compared to coculture N/AS at all time points recorded (Figure 4A, DIV13 *p* = 0.0456, DIV14 to DIV17 *p* < 0.0001). The mean firing rate was significantly lower in triculture N/AS/MG*^med^* compared to coculture N/AS at all time points recorded after DIV14 (Figure 4A, DIV15 *p* = 0.0024, DIV16 *p* < 0.0001, DIV17 *p* = 0.0048). Burst frequency was significantly lower in triculture N/AS/MG*^high^* compared to coculture N/AS at all time points recorded after DIV13 (Figure 4B, DIV14 *p* = 0.003, DIV15 to DIV17 *p* < 0.0001). Burst frequency was significantly lower in triculture N/AS/MG*^med^* compared to coculture N/AS at all time points recorded after DIV13 (Figure 4B, DIV14 *p* = 0.0023, DIV15 p = 0.0001, DIV16 to DIV17 *p* < 0.0001). Network burst frequency was significantly lower in triculture N/AS/MG*^high^* compared to coculture N/AS at all time points recorded after DIV13 (Figure 4C, DIV14 *p* = 0.0058, DIV15 *p* = 0.0001, DIV16 to DIV17 *p* < 0.0001). Network burst frequency was significantly lower in triculture N/AS/MG*^med^* compared to coculture N/AS at all time points recorded between DIV14 and DIV16 (Figure 4C, DIV14 *p* = 0.0112, DIV15 *p* = 0.0007, DIV16 *p* = 0.0004). Representative activity traces (raster plots) at DIV17 from coculture and tricultures show the dose-dependent inhibition of neuronal activity by microglia (Figures 4D–F).

### Neurons in triculture retain activity capacity despite microglial attenuation of electrophysiological metrics

**FIGURE 5:**
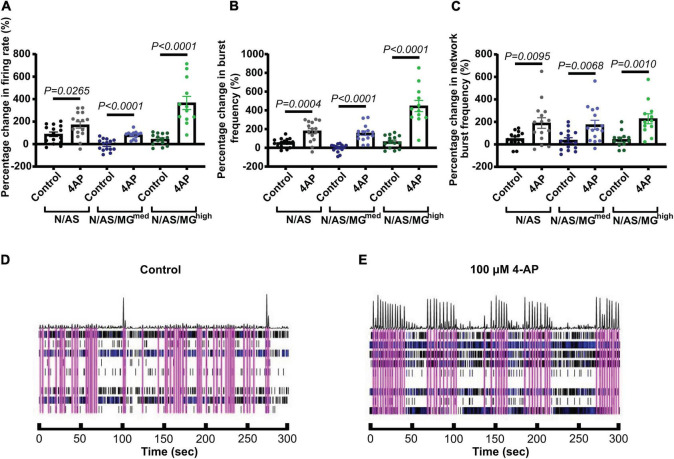
Treatment with 4-aminopyridine (4-AP) increases cortical neuronal activity in cocultures and tricultures. **(A)** On DIV17, cultures were treated with vehicle or 100 μM 4-AP. Scatter plot shows the percentage change in mean firing rate of neurons 1 h after treatment compared to their basal activity before drug treatment (N/AS *p* = 0.0265, N/AS/MG^med^ *p* < 0.0001, N/AS/MG^high^ *p* < 0.0001, Unpaired *t*-test). **(B)** On DIV17, cultures were treated with vehicle or 100 μM 4-AP. Scatter plot shows the percentage change in burst frequency of neurons 1 h after treatment compared to their basal activity before drug treatment (N/AS *p* = 0.0004, N/AS/MG^med^ *p* < 0.0001, N/AS/MG^high^ *p* < 0.0001, Unpaired *t*-test). **(C)** On DIV17, cultures were treated with vehicle or 100 μM 4-AP. Scatter plot shows the percentage change in network burst frequency of neurons 1 h after treatment compared to their basal activity before drug treatment (N/AS *p* = 0.0095, N/AS/MG^med^ *p* = 0.0068, N/AS/MG^high^ *p* = 0.001, Unpaired *t*-test). **(D,E)** Representative activity traces at DIV17 from triculture N/AS/MG^high^ treated with vehicle and 100 μM 4-AP. Black spikes at the top represent network activity while activity over time recorded on each of the eight electrodes is shown under. Blue = bursts. Pink = network bursts. Data shown as mean ± SEM and is representative of three independent experiments.

As neuronal activity was lower in the presence of microglia, we sought to investigate whether neurons were still functional and able to respond to stimulation or whether this capacity was disrupted in presence of microglia. Increased neuronal activity in response to the potassium channel blocker 4-AP has previously been characterised in other cell models (Parmentier et al., 2022). Neuronal activity in both cocultures and tricultures from three independent biological repeats was modulated with 4-AP (Figure 5). Treatment at DIV17 significantly increased the mean firing rate in cocultures and tricultures compared to their basal activity before drug treatment (Figure 5A, N/AS *p* = 0.0265, N/AS/MG*^med^ p* < 0.0001, N/AS/MG*^high^ p* < 0.0001). Treatment also increased burst frequency in cocultures and tricultures (Figure 5B, N/AS *p* = 0.0004, N/AS/MG*^med^ p* < 0.0001, N/AS/MG*^high^ p* < 0.0001). Network burst frequency was also increased by 4-AP treatment (Figure 5C, N/AS *p* = 0.0095, N/AS/MG*^med^ p* = 0.0068, N/AS/MG*^high^ p* = 0.001). The N/AS/MG*^high^* triculture displayed a larger increase in mean firing rate and burst frequency compared to N/AS or N/AS/MG*^med^*, whereas the percentage increase in network burst frequency was more consistent between cocultures and tricultures. Representative activity traces (raster plots) at DIV17 from N/AS/MG*^high^* tricultures treated with either vehicle or 4-AP show the increase in neuronal activity after drug addition (Figures 5D,E). In conclusion, modulation of cortical neuronal activity in cocultures and tricultures with 4-AP indicates that neurons in the triculture model remain functional and retain the capacity for neuronal activity in the presence of the microglia.

### Microglia reduce the densities of spines and the postsynaptic density scaffold protein Homer1 in secondary dendrites

**FIGURE 6:**
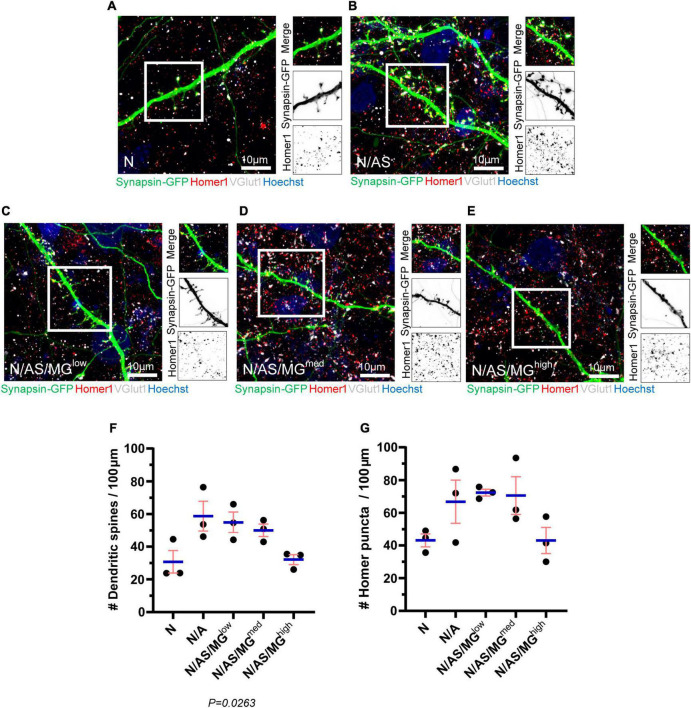
Spine density and Homer1 density are reduced in neuronal cultures and tricultures compared to cocultures. **(A–E)** Representative immunostaining images of dendrites used for quantification of spines and Homer1 density showing Synapsin-GFP, Homer1, and VGlut1 expression at DIV17 in neuronal cultures, cocultures, and tricultures. Image shows staining of Synapsin-GFP (green), Homer1 (red), VGlut1 (white), and Hoechst (blue). Scale bar = 10 μm. White frame = field of view of merge. **(F,G)** Analysis performed on neuronal cultures, cocultures, and tricultures to quantify the number of spines and Homer1 puncta using Imaris software. The difference in spine densities between the five culture conditions is statistically significant (*p* = 0.0263, one-way ANOVA). The difference in Homer1 densities between the five culture conditions is not statistically significant (*p* = 0.0839, one-way ANOVA). Data shown as mean ± SEM and is representative of three independent experiments.

Following our finding that microglia suppress spontaneous neuronal activity, we further investigated the impact of microglia on neurons in the triculture model. Neuronal cultures, cocultures, and tricultures were transfected with Synapsin-GFP (Figure 1A) using Neuromag magnetofection, before fixing on DIV17 and immunostaining for the presynaptic protein VGlut1 and postsynaptic protein Homer1 (Figures 6A–E). The Neuromag method has been demonstrated to achieve up to 30% transfection efficiency in primary cortical neurons (Wang et al., 2014), and in one case was optimised to reach efficiencies higher than 45% in primary motor neurons without altering survival or morphology (Fallini et al., 2010). In our study, magnetofection was used for sparse transfection in order to facilitate the visualisation of individual neurons, dendrites and spines. After fixing, neurons appeared healthy and the rate of transfection was 4.43 ± 0.64% (data not shown). This is consistent with a separate study in which Neuromag magnetofection on DIV7 achieved a transfection rate of 3.2 ± 1.2% in primary cortical neurons and no differences were noted between magnetofected or control cultures with respect to morphology, percentage of live cells, number of neurons or percentage of pyknotic nuclei (Evans et al., 2017).

Analysis of the transfected neurons shows that dendritic spine density is reduced in neuronal cultures and tricultures compared to cocultures. The trend indicates that microglia reduce spine density in a dose-dependent manner, with N/AS/MG*^high^* tricultures displaying similar spine densities to that of the neuronal cultures. The difference in spine densities between the five culture conditions is statistically significant (*p* = 0.0263; Figure 6F). While not significant, the density of Homer1 puncta in neuronal cultures, cocultures, and tricultures follows a similar trend, albeit with more variation between biological repeats (*p* = 0.0839; Figure 6G). The percentage of Homer1 puncta co-localised with VGlut1 does not indicate a clear trend (data not shown).

## Discussion

We developed an *in vitro* rat triculture model of neurons, astrocytes and microglia that is suitable for pharmacological modulation. The triculture is established by adding exogenous microglia to cocultures of neurons and astrocytes and culturing the cells in a triculture medium supplemented with TGF-β, IL-34, and cholesterol. These three components were chosen as they are the key factors sufficient for promoting microglial survival under serum-free conditions (Bohlen et al., 2017) and have been used previously to generate a primary triculture model (Goshi et al., 2020). Immunostaining shows that there is a healthy population of neurons and astrocytes in both the cocultures and tricultures, and the number of neurons is not affected by the presence of the microglia. The number of astrocytes appears to be slightly reduced in the triculture with the highest ratio of microglia. While it is possible that only expression of Sox9 is affected, lower numbers of astrocytes have previously been observed in tricultures compared to cocultures by quantifying the number of nuclei co-localised with glial fibrillary acidic protein (GFAP) (Goshi et al., 2020). One possible explanation is that microglia express TGF-β1, which has been shown to inhibit astrocyte proliferation (Lindholm et al., 1992; De Groot et al., 1999). Further analysis indicates that the percentage of homeostatic microglia decreases dose-dependently as the number of microglia increases. Therefore, tricultures with higher numbers of microglia contain a microglia population that is proportionally more reactive.

By using MEA as an endpoint to investigate glia-mediated regulation of cortical neuronal activity, we have demonstrated that neuronal cultures exhibit lower basal activity than cocultures of neurons and astrocytes, which is consistent with the reduced spine and Homer1 densities displayed in our neuronal cultures. Previous studies have also shown that astrocytes increase the number of mature, functional synapses on neurons (Ullian et al., 2001). This is thought to occur via the secretion of factors such as thrombospondins which are known to promote synaptogenesis (Christopherson et al., 2005). We also demonstrated that tricultures exhibit lower basal activity than cocultures and that microglia suppress neuronal activity in a dose-dependent manner. Pharmacological modulation of these cultures with 4-AP indicates that neurons remain functional in the presence of the microglia even if their basal activity is lower. Microglia have previously been shown to regulate neuronal activity. Neurons generate adenosine triphosphate (ATP), which attracts microglial processes to neurons with high levels of activity. In zebrafish this microglia-neuron contact requires the small Rho GTPase Rac in microglia, and results in reduced activity in the contacted neurons (Li et al., 2013). Microglia also respond to neuronal activation by suppressing neuronal firing and the associated seizure response in mice. The mechanism of suppression is dependent on the microglial sensing of ATP and its subsequent conversion into adenosine (Badimon et al., 2020). Another possible mechanism for the downregulation of neuronal activity in the triculture model is complement-mediated synapse elimination. This hypothesis is supported by the fact that microglia reduce the densities of spines and Homer1 puncta in a dose-dependent manner in our triculture model. The formation of mature neural circuits requires activity-dependent pruning of inappropriate, less active synapses, and this process occurs via activation of C3 receptors on microglia, triggering phagocytosis (Stevens et al., 2007). The complement-dependent synaptic pruning that occurs during development is thought to be reactivated and contribute to synapse loss in AD (Hong et al., 2016). Microglia may also reduce synapse numbers via a non-phagocytic mechanism. In the retinogeniculate circuit, microglia expressing TNF-related weak inducer of apoptosis (TWEAK) facilitate synapse elimination through the neuronal receptor Fibroblast growth factor-inducible 14 (Fn14) (Cheadle et al., 2020).

It is important to note that while our model can provide insights into the crosstalk between microglia, astrocytes and neurons, it may not fully recapitulate the interactions involved in human disease. Cultures generated from rodents are an important research tool and have contributed heavily to our understanding of disease mechanisms. In many respects they mirror human biology closely, and the genes identified as being associated with inherited disease in humans are highly conserved in the rat genome (Huang et al., 2004). However, understanding species-specific differences in the neuroinflammatory response remains a major challenge in the field and there are recognised limitations in using mouse models to mimic human disease, specifically neurodegeneration (Arber et al., 2017). Further development of our model could incorporate cell types of other species. A triculture system of human induced pluripotent stem cell (hiPSC)-derived microglia, astrocytes and neurons containing the APPSWE+/+ mutation has been used to model AD and showed increased production of the C3 protein due to microglia initiating reciprocal signalling with astrocytes (Guttikonda et al., 2021). While human induced pluripotent stem cells (hiPSCs) provide a valuable tool for disease modelling using physiologically relevant cells, they can prove challenging when attempting to generate a robust, reproducible culturing platform for drug discovery. Automated technology has been utilised to eliminate the variation that can arise during differentiation and produce consistent and long-term tricultures of hiPSC neurons, astrocytes, and microglia. This model displays many pathological features of AD such as Aβ plaques, phospho-tau induction and synapse loss, and has been used to investigate the mechanism of action of anti-Aβ antibodies (Bassil et al., 2021). Mixed species cultures provide additional opportunities to probe the roles of individual cell types within a more complex model. RNA-sequencing on cultures combining rat, mouse and human cells allows for individual cell-type transcriptomes to be profiled. This approach has been used to show that microglial ramification is controlled by the combined effects of neurons and astrocytes via TGF-β2 signalling (Baxter et al., 2021).

Most AD drug targets which displayed favourable outcomes in biochemical, cell culture and AD transgenic models have failed to prove effective in clinical trials (De Strooper, 2014). One possible explanation for this is the limited ability of these models to mimic human disease. In 2D cell culture models of AD, secreted Aβ may diffuse into the cell culture media and be removed during media changes (D’Avanzo et al., 2015). Recent advances in stem cell and 3D culture technologies have made it possible to generate novel models that more closely recapitulate AD pathology in the human brain. A neural stem-cell-derived 3D culture system generated using Matrigel-based technology behaves more like brain tissue and provides a closed environment promoting aggregation of Aβ. In this model, familial AD mutations in β*-amyloid precursor protein* (APP) and *presenilin 1* (PSEN1) induce robust extracellular deposition of Aβ, including Aβ plaques, and aggregates of phosphorylated tau in the soma and neurites (Choi et al., 2014). While these 3D cell culture models provide a promising, more physiologically relevant platform for high-throughput drug screening, neurospheroids can be sensitive to small variations and difficult to manipulate experimentally. Recently, however, a platform has been developed using new tools and stem cell engineering to reproducibly generate neurospheroids inside a 96-well cell culture array plate with 1,536 microwells. Unlike other brain organoids, in this model the dendrites extend outward which allows for the formation of networks (Jorfi et al., 2018).

As neuron-glia interactions become increasingly recognised to play a critical role in the pathogenesis of AD, the development of complex models and endpoints allowing for modulation of glia within a system has become more important for drug discovery. While 3D models allow for more complex modelling of disease, the 2D model we describe here may provide a more realistic alternative for many studies as it is easier to generate and can be used to measure physiologically relevant endpoints. Numerous studies have targeted glia in models of neurodegenerative diseases. For example, pharmacological blockade of Connexin 43 (Cx43) in amyotrophic lateral sclerosis (ALS) astrocytes has been shown to provide neuroprotection of motor neurons and reduce neuronal hyperexcitability in a coculture model (Almad et al., 2022). Inhibition of astrocytic α2-Na+/K+ adenosine triphosphatase (α2-NKA) in a mouse model of tauopathy suppresses neuroinflammation, and its knockdown halts the progression of tau pathology (Mann et al., 2022). Blocking of the astrocytic calcium channel Transient Receptor Potential Ankyrin 1 (TRPA1) or the enzyme epoxide hydrolase, which is predominantly expressed by astrocytes, have both been shown to normalise astrocytic activity, prevent neuronal dysfunction and improve cognitive function in AD mouse models (Ghosh et al., 2020; Paumier et al., 2022). Triggering receptor expressed on myeloid cells 2 (TREM2) is essential for the transition of homeostatic microglia to a disease-associated state. Monoclonal antibody-mediated stabilisation and activation of TREM2 on the cell surface reduces AD-related pathology in a mouse model and reduces levels of the homeostatic marker P2Y12 receptor (P2RY12), suggesting that driving microglia toward a disease-associated state might provide a protective function (Schlepckow et al., 2020). Activation of TREM2 in an AD mouse model modulates the microglial inflammatory response, reduces neurite dystrophy and restores the behavioural changes associated with Aβ pathology (Wang et al., 2020).

In summary, our neuron, astrocyte and microglia triculture model provides a robust and reliable tool for studying the role of glia-neuron crosstalk in the regulation of neuronal activity, and how this is impacted by neuroinflammatory processes in disease. The use of this model in combination with MEA technology allows for pharmacological manipulation of the system in a high-throughput manner and has the potential to be used for target validation and drug screening.

## Data availability statement

The original contributions presented in this study are included in the article/supplementary material, further inquiries can be directed to the corresponding author.

## Ethics statement

The animal study was reviewed and approved by the UCL Ethics Committee.

## Author contributions

LP, DL, and SJ designed the experiments. LP, DL, NA, SI, CN, and LG performed the experiments. LP and SJ wrote the manuscript with input from all authors. All authors discussed the results, contributed to the article, and approved the submitted version.

## Footnotes

1
http://n2t.net/addgene:114215
